# Genome-wide association study for circulating fibroblast growth factor 21 and 23

**DOI:** 10.1038/s41598-020-71569-8

**Published:** 2020-09-03

**Authors:** Gwo-Tsann Chuang, Pi-Hua Liu, Tsui-Wei Chyan, Chen-Hao Huang, Yu-Yao Huang, Chia-Hung Lin, Jou-Wei Lin, Chih-Neng Hsu, Ru-Yi Tsai, Meng-Lun Hsieh, Hsiao-Lin Lee, Wei-shun Yang, Cassianne Robinson-Cohen, Chia-Ni Hsiung, Chen-Yang Shen, Yi-Cheng Chang

**Affiliations:** 1 Department of Pediatrics, National Taiwan University Hospital, College of Medicine, National Taiwan University, Taipei, Taiwan, ROC; 2 Graduate Institute of Medical Genomics and Proteomics, National Taiwan University, 5F, No.2, Xuzhou Rd., Zhongzheng Dist., Taipei, 100 Taiwan, ROC; 3 Clinical Informatics and Medical Statistics Research Center, College of Medicine, Chang Gung University, Taoyuan, Taiwan, ROC; 4 Division of Endocrinology and Metabolism, Department of Internal Medicine, Chang Gung Memorial Hospital At Linkou, Taoyuan, Taiwan, ROC; 5 Institute of Molecular Medicine, College of Medicine, National Taiwan University, Taipei, Taiwan, ROC; 6 Department of Medical Nutrition Therapy, Chang Gung Memorial Hospital At Linkou, Taoyuan, Taiwan, ROC; 7 Department of Chinese Medicine, College of Medicine, Chang Gung University, Taoyuan, Taiwan, ROC; 8 Cardiovascular Center, National Taiwan University Hospital Yun-Lin Branch, Taipei, Taiwan, ROC; 9 Department of Internal Medicine, College of Medicine, National Taiwan University, Taipei, Taiwan, ROC; 10 Division of Nephrology, Department of Medicine, Vanderbilt University Medical Center, Nashville, TN USA; 11 Data Science Statistical Cooperation Center, Institute of Statistical Science, Academia Sinica, Taipei, Taiwan, ROC; 12 Institute of Biomedical Sciences, Academia Sinica, Taipei, Taiwan, ROC; 13 College of Public Health, China Medical University, Taichung, Taiwan, ROC

**Keywords:** Genetics, Endocrinology

## Abstract

Fibroblast growth factors (FGFs) 21 and 23 are recently identified hormones regulating metabolism of glucose, lipid, phosphate and vitamin D. Here we conducted a genome-wide association study (GWAS) for circulating FGF21 and FGF23 concentrations to identify their genetic determinants. We enrolled 5,000 participants from Taiwan Biobank for this GWAS. After excluding participants with diabetes mellitus and quality control, association of single nucleotide polymorphisms (SNPs) with log-transformed FGF21 and FGF23 serum concentrations adjusted for age, sex and principal components of ancestry were analyzed. A second model additionally adjusted for body mass index (BMI) and a third model additionally adjusted for BMI and estimated glomerular filtration rate (eGFR) were used. A total of 4,201 participants underwent GWAS analysis. rs67327215, located within *RGS6* (a gene involved in fatty acid synthesis), and two other SNPs (rs12565114 and rs9520257, located between *PHC2-ZSCAN20* and *ARGLU1-FAM155A* respectively) showed suggestive associations with serum FGF21 level (*P* = 6.66 × 10^–7^, 6.00 × 10^–7^ and 6.11 × 10^–7^ respectively). The SNPs rs17111495 and rs17843626 were significantly associated with FGF23 level, with the former near *PCSK9* gene and the latter near *HLA-DQA1* gene (*P* = 1.04 × 10^–10^ and 1.80 × 10^–8^ respectively). SNP rs2798631, located within the *TGFB2* gene, was suggestively associated with serum FGF23 level (*P* = 4.97 × 10^–7^). Additional adjustment for BMI yielded similar results. For FGF23, further adjustment for eGFR had similar results. We conducted the first GWAS of circulating FGF21 levels to date. Novel candidate genetic loci associated with circulating FGF21 or FGF23 levels were found. Further replication and functional studies are needed to support our findings.

## Introduction

Fibroblast growth factor (FGF) superfamily includes 22 family members. Except for the endocrine FGF19, FGF21, and FGF23, other family members act as autocrine or paracrine factors^1^. Recent evidence showed that FGF21 exerts metabolic effects by acting both centrally and peripherally, mostly in the liver, adipose tissue and pancreas. In prolonged starvation, FGF21 is secreted by the liver to promote gluconeogenesis, increases hepatic fatty acid oxidation and ketogenesis^2^, enhances secretion of glucocorticoid, and reduces behaviors that waste energy such as ovulation^3^. In contrast, in fed status, FGF21 promotes glucose uptake by fat cells and increase heat production of brown fat^4^. In the pancreatic islet, FGF21 preserves the survival of beta-cell and restores insulin synthesis under the stress of nutrient excess^5^. Of note, FGF21 acts on the brain to induce sympathetic nerve activity, thereby increasing energy expenditure and promoting weight loss^6^. FGF21 is regulated by both peroxisome proliferator-activated receptor (PPAR) γ and PPARα and is an important mediator of the downstream effects of PPARγ and PPARα^6,7^. FGF21 is currently a drug target for treating lipid disorders and fatty liver^8^.

FGF23 is the physiological regulator of phosphate and vitamin D serum levels. FGF23 lowers apical membrane expression of sodium-dependent phosphate co-transporter 2A and sodium-dependent phosphate co-transporter 2C in the kidney, both of which primarily mediate renal tubular phosphate reabsorption. Furthermore, FGF23 lowers serum 1,25-dihydroxyvitamin D_3_ (1,25(OH)_2_D_3_) levels, thereby preventing hyperphosphatemia and hypervitaminosis^1,9^. FGF23 has structure and biological features similar to those of FGF19 and FGF21, which were known to regulate glucose and lipid metabolism^10^. FGF23 level was also found to be associated with obesity and dyslipidemia in studies of elderly cohorts^11^. FGF23 is also an important drug target for treating phosphate disorder and bone disease^12^. Robinson-Cohen et al. performed a genome-wide association study (GWAS) of circulating FGF23 concentrations among people of European ancestry, and found five genome-wide significant loci^13^.

To understand better the regulation of circulating FGF21 and FGF23 levels, we performed a GWAS to identify genetic determinants of circulating FGF21 and FGF23 levels in Taiwanese population.

## Results

Of the 5,000 subjects enrolled from Taiwan Biobank, one withdrew from the study, 559 were excluded due to their having diabetes mellitus, and 239 were excluded by quality control procedures. A total of 617,073 genotyped autosomal SNPs remained. After imputation and post-imputation quality control, 7,897,704 SNPs remained. We performed GWAS analysis in the remaining 4,201 subjects, where log-transformed FGF21 or FGF23 level was the quantitative phenotype. Study characteristics are shown in Table 1.

**Table 1:** Study characteristics of participants.

Characteristic	
Total participants (N)	4,201
Age, year	49.54 ± 10.82
Males (%)	1,446 (34.42)
BMI, kg/m^2^	24.32 ± 6.67
Serum glucose, mg/dl	93.01 ± 8.26
eGFR (MDRD), ml/min/1.73 m^2^	106.80 ± 24.98
FGF21, ng/ml	0.16 (0.10–0.27)
FGF23, ng/ml	0.36 (0.11–1.74)

Data are mean ± SD, median (IQR) or number (%), as appropriate. Age is at specimen collection.

*BMI* body mass index; *MDRD* modification of diet in renal disease equation.

**Figure 1:**
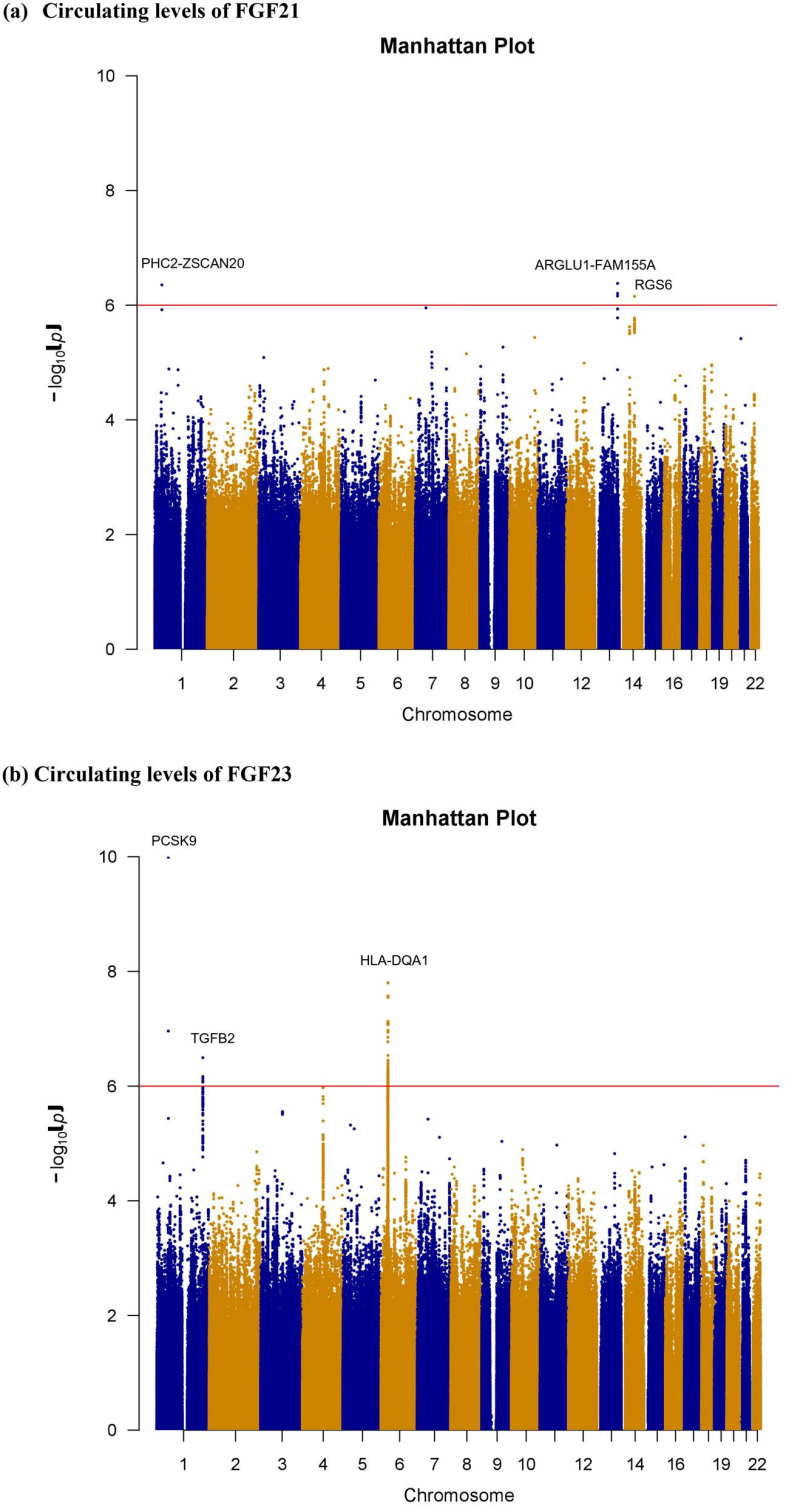
Manhattan plot of the GWAS results for FGF21. SNPs are plotted on the x axis according to their chromosome position against association with (**a**) log FGF21 or (**b**) log FGF23 on the y axis. The red horizontal line represents the suggestive threshold of P = 1.0 × 10^–6^.

**Figure 2:**
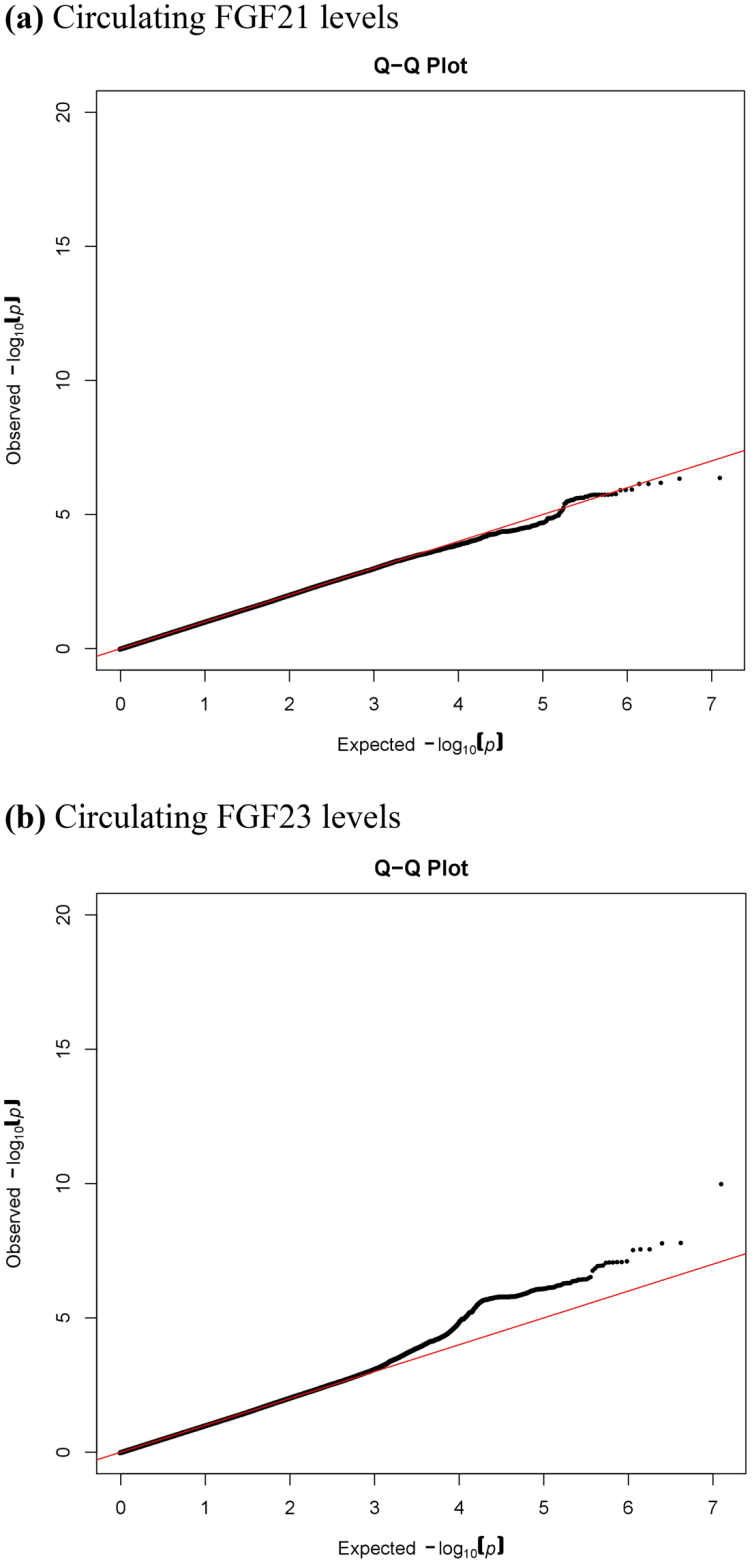
Quantile–quantile plots of (**a**) log FGF21 and (**b**) log FGF23.

**Figure 3:**
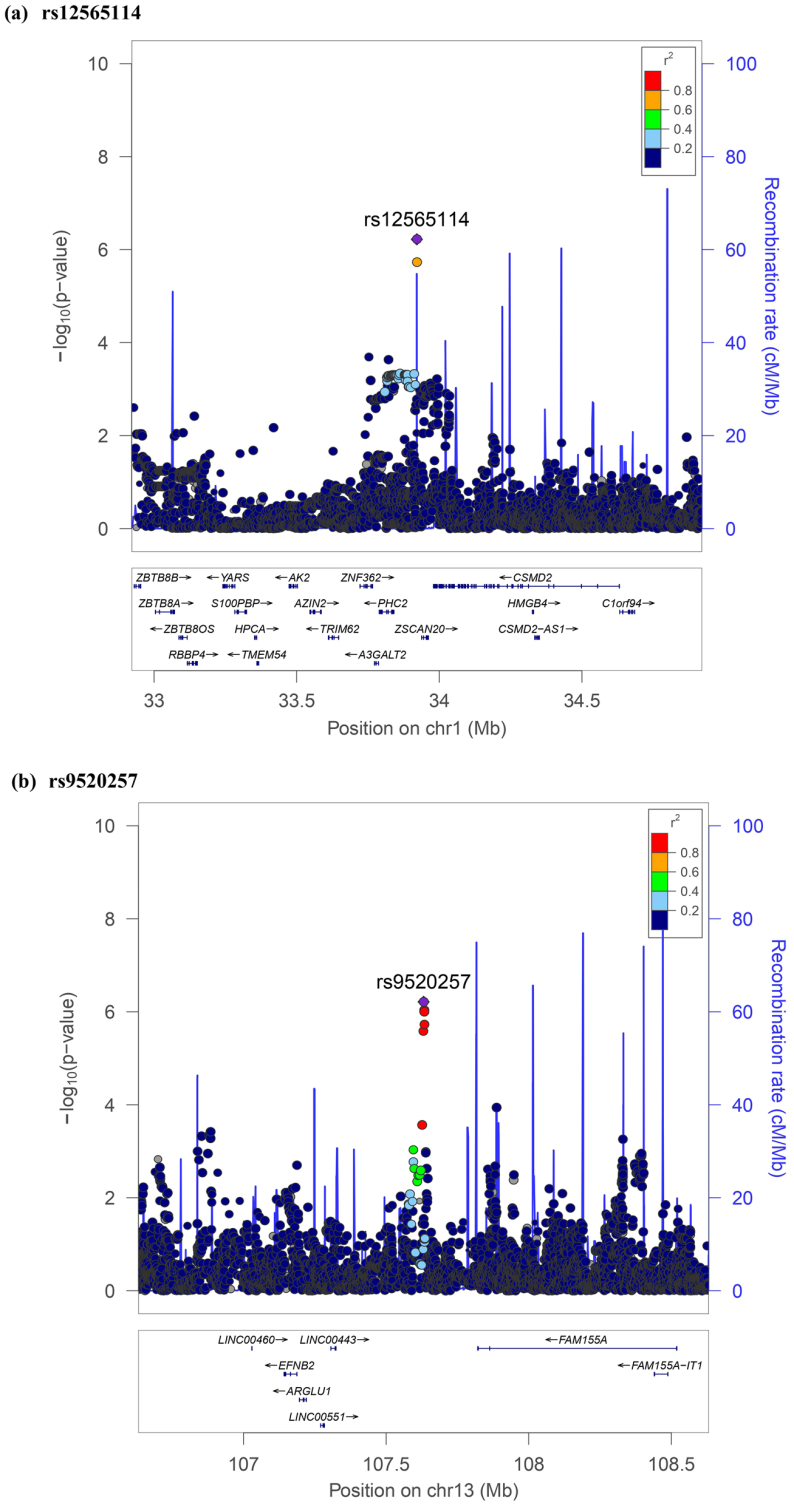

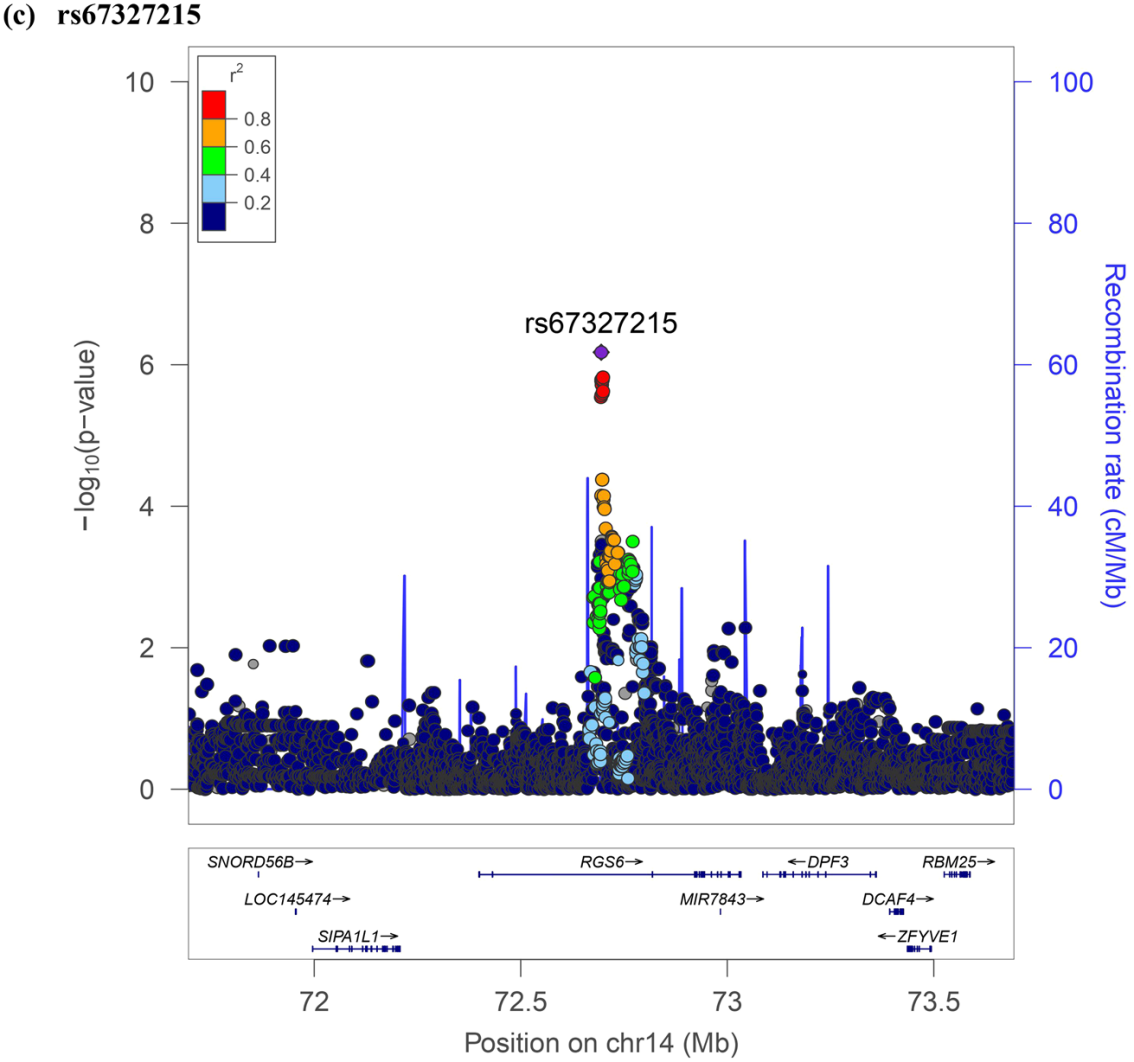
Regional association plots of log FGF21.

**Figure 4:**
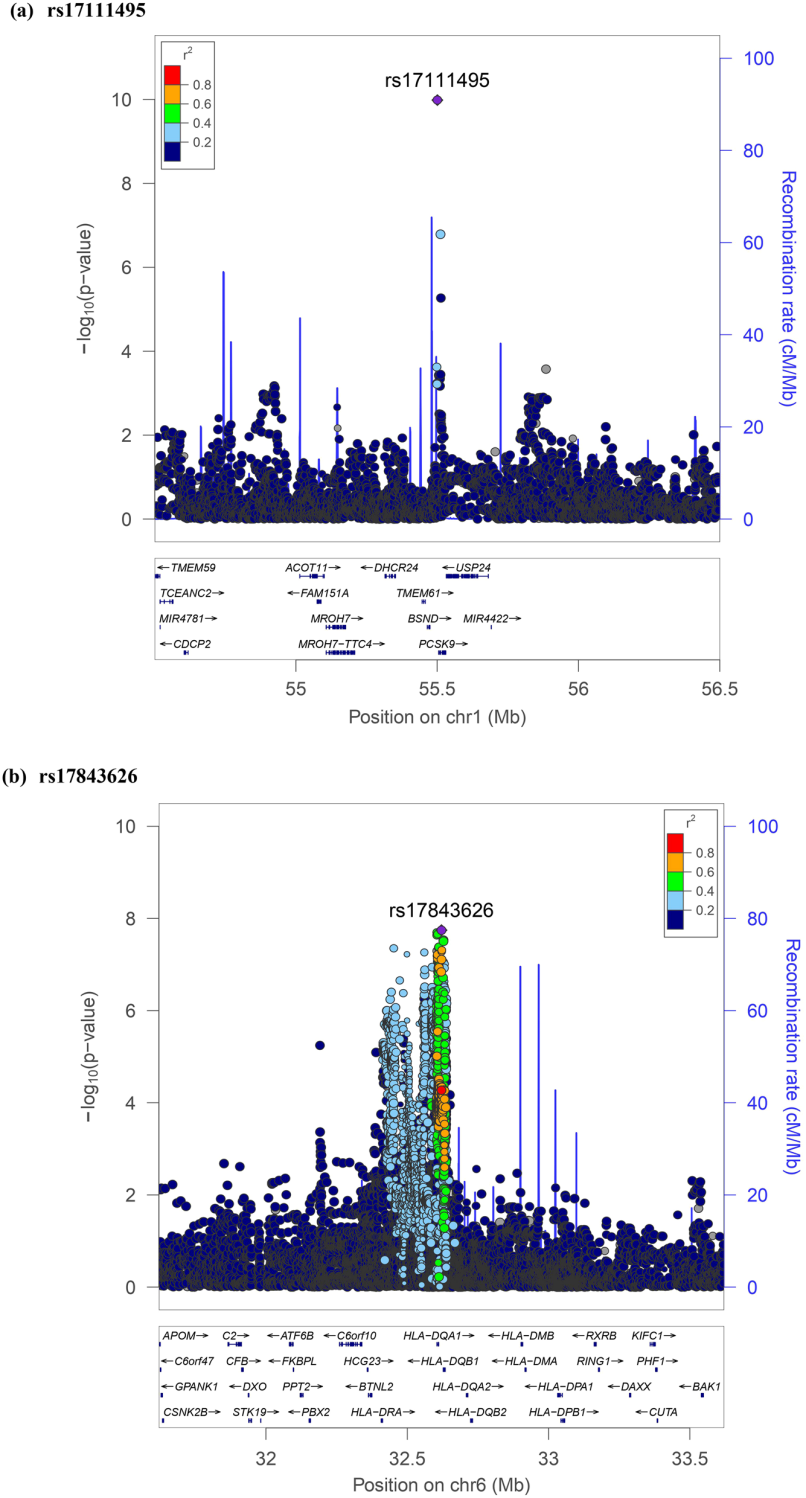

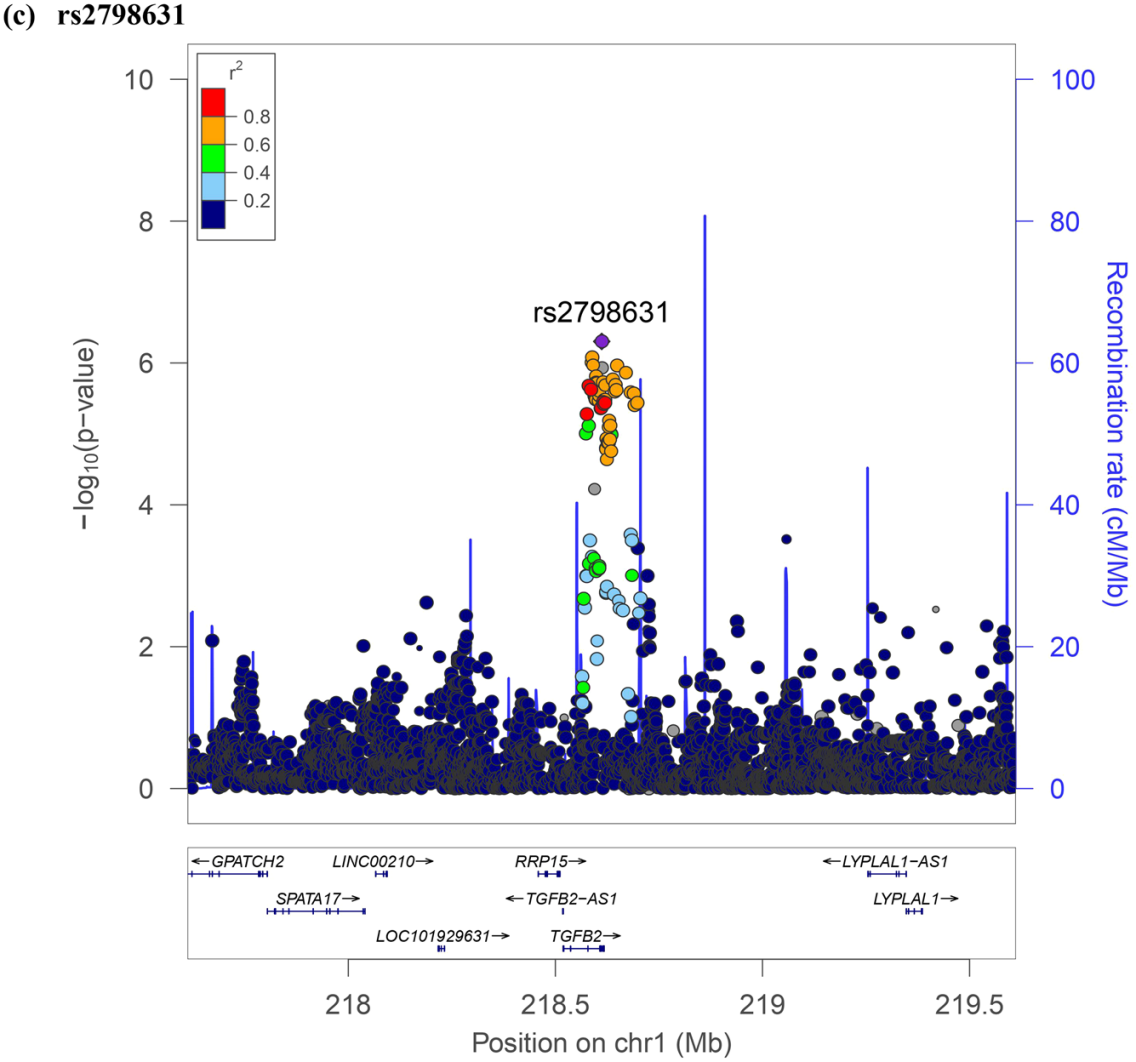
Regional association plots of log FGF23.

The results of association testing for FGF21 and FGF23 are listed, showing SNP with strongest association in each region (Tables 2 and 3, respectively). Manhattan plots and quantile–quantile plots of log-transformed FGF21 and FGF23 are shown in Figs. 1 and 2, respectively. Regional GWAS plots of each SNP are shown in Figs 3 and 4. For FGF21, no significantly associated SNP is found. One suggestive locus is located at the intergenic region between *PHC2* and *ZSCAN20* genes on chromosome 1 (rs12565114; *P* = 6.00 × 10^–7^, Fig. 3a), another is between *ARGLU1* and *FAM155A* genes on chromosome 13 (rs9520257; *P* = 6.11 × 10^–7^, Fig. 3b), and the other is within the *RGS6* gene on chromosome 14 (rs67327215; *P* = 6.66 × 10^–7^, Fig. 3c). For FGF23, two significant loci are found. One locus is near gene *PCSK9* on chromosome 1 (rs17111495; *P* = 1.04 × 10^–10^, Fig. 4a), and the other is near the *HLA-DQA1* gene on chromosome 6 (rs17843626; *P* = 1.80 × 10^–8^, Fig. 4b). There is also a suggestive locus within the *TGFB2* gene on chromosome 1 (rs2798631; *P* = 4.97 × 10^–7^, Fig. 4c). The array-based heritability estimates for FGF21 and FGF23 were 0.1171 and 0.1104 respectively. Proportion of variance in serum FGF21 level explained by rs12565114, rs9520257 and rs67327215 were 0.5%, 0.6% and 0.5% respectively; for serum FGF23 level, proportion of variance explained by rs17111495, rs2798631 and rs17843626 were 1.0%, 0.6% and 0.7% respectively.

**Table 2:** Top genetic polymorphisms associated with log-transformed FGF21 level.

SNP	Chr	Position	Nearest gene	FGF21 Increasing allele	Other allele	FGF21 increasing allele frequency	Model 1		Model 2	
							P value	Beta (SEM)	P value	Beta (SEM)
rs12565114	1	33,921,202	*PHC2-ZSCAN20*	G	A	0.69	6.00 × 10^–7^	0.048 (0.010)	4.69 × 10^–7^	0.049 (0.010)
rs9520257	13	107,631,394	*ARGLU1-FAM155A*	C	T	0.85	6.11 × 10^–7^	0.062 (0.012)	3.96 × 10^–7^	0.063 (0.012)
rs67327215	14	72,694,874	*RGS6*	G	A	0.62	6.66 × 10^–7^	0.046 (0.009)	6.93 × 10^–7^	0.046 (0.009)

Model 1 was adjusted for age, sex and the first ten principal components of ancestry. Model 2 was additionally adjusted for BMI. *SNP* single nucleotide polymorphism; *Chr* chromosome. rs12565114 and rs9520257 are genotyped. rs67327215 is imputed with high info score (0.984).

**Table 3:** Top genetic polymorphisms associated with log-transformed FGF23 level.

SNP	Chr	Position	Nearest gene	FGF23 Increasing allele	Other allele	FGF23 increasing allele frequency	Model 1		Model 2		Model 3	
							P value	Beta (SEM)	P value	Beta (SEM)	P value	Beta (SEM)
rs17111495	1	55,500,706	*PCSK9*	G	C	0.98	1.04 × 10^–10^	0.463 (0.071)	1.57 × 10^–10^	0.458 (0.071)	1.55 × 10^–10^	0.458 (0.071)
rs17843626	6	32,621,013	*HLA-DQA1*	G	A	0.49	1.80 × 10^–8^	0.117 (0.021)	1.70 × 10^–8^	0.117 (0.021)	1.75 × 10^–8^	0.117 (0.021)
rs2798631	1	218,611,878	*TGFB2*	G	A	0.75	4.97 × 10^–7^	0.115 (0.023)	3.73 × 10^–7^	0.116 (0.023)	3.71 × 10^–7^	0.116 (0.023)

Model 1 was adjusted for age, sex and the first ten principal components of ancestry. Model 2 was additionally adjusted for BMI. Model 3 was additionally adjusted for BMI and eGFR. *SNP* single nucleotide polymorphism; *Chr* chromosome. rs17111495, rs2798631 and rs17843626 are imputed with high info scores (0.809, 0.997 and 0.990 respectively).

Further excluding two more subjects with severe renal impairment (eGFR < 30 ml/min/1.73m^2^) did not alter the results of top SNPs associated with FGF23 level (Supplementary Table S1).

We looked for the association of the five SNPs identified to be associated with serum FGF23 levels by Robinson-Cohen et al.^13^ in the European population but failed to found significant association with the FGF23 level in our cohort (Supplementary Table S2). In view of low minor allele frequency of rs17216707, we performed gene-based association analysis using MAGMA v1.07b^14^ and still found no significant association (*CYP24A1*, *P* = 0.18). The regional plots regarding association with serum FGF23 levels in Taiwan Biobank at the five loci identified previously by Robinson-Cohen and colleagues are shown in Supplementary Fig. S1a–e. We also performed meta-analysis pooling our results and those reported by Robinson-Cohen et al.^13^ using METAL^15^ but the results were not significant as shown in Supplementary Table S3 (*P* values were 0.00070, 0.0010 and 0.00029 for rs17111495, rs2798631 and rs17843626, respectively).

## Discussion

In this GWAS for evaluating both circulating FGF21 and FGF23 levels, we identified several loci for each trait that showed associations. SNP rs67327215, associated with FGF21 level, is located within *RGS6* (Regulator of G protein signaling 6). RGS6 belongs to the R7 subfamily of RGS proteins^16^ and is expressed in a variety of organs and tissues, including the central nervous system and liver^17^. RGS proteins regulate G protein-coupled receptor-initiated signaling by acting as GTPase-activating proteins for Gα subunits, which hydrolyze GTP and restore the inactive Gα _GDP_ βγ heterotrimer^18^. A previous study has shown *Rgs16* knockout mice expressing increased *Fgf21* expression in the liver while overexpression of *Rgs16* decreased *Fgf21* expression. It was postulated that RGS16 inhibits Gα_i_/Gα_q_-mediated fatty acid oxidation, thereby decreasing the induced *Fgf21* expression by peroxisome proliferator-activated receptor alpha (PPARα)^19^. RGS6 knockout mice revealed lower mRNA levels of PPARα and PPARγ^20^. These data support that RGS6 may also act as a regulator of circulating FGF21 levels through fatty acid synthesis and oxidation.

The SNPs rs12565114 and rs9520257, also suggestively associated with FGF21 level, are intergenic SNPs on chromosome 1 and 13 respectively. rs12565114 is located between *PHC2* (Polyhomeotic Homolog 2) and *ZSCAN20* (Zinc Finger And SCAN Domain Containing 20) genes. PHC2 is associated with conventional angiosarcoma^21^. ZSCAN20 is associated with diabetic neuropathic pain^22^. rs9520257 is located between gene *ARGLU1* (Arginine And Glutamate Rich 1) and *FAM155A* (Family With Sequence Similarity 155 Member A) genes. ARGLU1 acts in cooperation with MED1 (Mediator Complex Subunit 1) and is required for estrogen-dependent gene transcription and breast cancer cell growth^23^. FAM155A is associated with diverticulitis^24^. None of the genes above were related to FGF21 according to our current understanding. There might be microRNAs or long non-coding RNAs encoded in these intergenic regions affecting FGF21 levels.

SNP rs17111495, strongly associated with FGF23 levels, is located upstream of *PCSK9* (Proprotein convertase subtilisin/kexin type 9). PCSK9 is a member of proprotein convertase family PCSK (subtilisin-like proprotein convertases previously) and is synthesized as a soluble zymogen that undergoes autocatalytic intramolecular processing in the endoplasmic reticulum^25^. FGF23 is inactivated when being cleaved intracellularly by PCSK at the minimum consensus sequence RHTR^179^ between Arg^179^ and Ser^180^^26,27^. Autosomal dominant hypophosphatemic rickets is caused by gain of function mutations in FGF23 that renders it resistant to PCSK cleaving at site RHTR^179^, thus resulting in elevated circulating FGF23 level and the consequent renal phosphate wasting, rickets, and osteomalacia^26^. Hyperphosphatemic familial tumoral calcinosis due to GALNT3 mutation involves deficient O-glycosylation of the threonine residue in the RTHR^179^ proprotein convertase-processing site, thus favoring intracellular degradation of intact FGF23 by PCSK and the resulting reduced phosphate renal excretion^28^. Collectively, previous studies and our findings suggest that PCSK is a critical regulator of FGF23 secretion.

SNP rs17843626, significantly associated with circulating FGF23 levels, is located between *HLA-DQA1* and *HLA-DQB1* (Human Leukocyte Antigen, Class II, DQ Alpha 1 and Beta 1). In general, these HLA class II loci are known to be associated with autoimmune diseases such as type 1 diabetes, multiple sclerosis and rheumatoid arthritis^29^. No literature has reported association between these loci and FGF23 level or its regulators, parathyroid hormone and vitamin D. This is also a new finding.

SNP rs2798631, suggestively associated with circulating FGF23 levels, is located within *TGFB2* (Transforming growth factor beta-2), which is involved in cell proliferation, differentiation, inflammation and apoptosis. A recent study has demonstrated that TGF-β2 directly enhanced FGF23 production in rat osteoblast-like cells through increasing calcium entry^30^. 1,25(OH)_2_D_3_, the active vitamin D metabolite which induces FGF23 production, also inhibits TGF-β downstream signaling^31^. Downstream TGF-β and 1,25(OH)_2_D_3_ signaling are characterized by intense crosstalk^32^. The expression of FGF23 by both modulators also seemed to be subjective to this crosstalk^33^.

For more comprehensive bioinformatic annotations of these top SNPs, we used GRASP v2.0 to search for documented associations between these SNPs and phenotypes^34^. One study showed association between rs12565114 and schizophrenia^35^. Several studies demonstrated association between rs17111495 and serum lipid levels^36,37^. rs2798631 was associated with height^38^, refractive error^39^ and Parkinson’s disease^40^. There were no results for rs9520257, rs67327215 and rs17843626.

The absolute circulating FGF23 levels measured in this study were different from that obtained by Robinson-Cohen et al. This study used Duoset ELISA Development kit (R&D system, Inc.) to measure intact FGF23 levels; and the median intact FGF23 level in the studied population was 360 (110–1,740) pg/ml (expressed as median and interquartile range). For published researches using this ELISA kit, the median of intact FGF23 level was 269.9 (109.2–1,014.0) pg/ml in Chinese renal transplant donors^41^, and 150 (46–583) pg/ml in Mexicans with normal kidney function^42^. In contrary, Robinson-Cohen et al. used a different ELISA kit (Kainos Laboratories, Inc., Tokyo, Japan), and the population for their study was of European ancestry. Their mean intact FGF23 level was around 40–50 pg/ml^13^. The variations in FGF23 level obtained may be attributed to ethnicity of the studied populations and different ELISA kits used.

Our study has some unique strength and some limitations. It is the first known study identifying suggestive genetic determinants of circulating FGF21 level. We also evaluated SNPs associated with circulating FGF23 level from a population of ancestry different from those studied in previous GWAS, and novel candidate loci were found.

As mentioned above, our findings differed from those of Robinson-Cohen et al.^13^, possibly due to differences in ethnicity, differences in gene-environment and gene–gene interactions, and our relatively small sample size. As rs17216707 minor allele frequency was low in our cohort, gene-based association analysis was conducted as an alternative way in effort to replicate the findings but the result was not significant. A meta-analysis conducted on both this study and that of Robinson-Cohen et al. found no statistically significant results either.

Regarding the lack of replication in an external cohort of the study findings, we searched for GWAS summary statistics of circulating FGF21 or FGF23 phenotypes in UK Biobank and Biobank Japan but found them unavailable. Further replication may be needed in the future.

In conclusion, this study is the first GWAS on circulating FGF21 level to date. Novel candidate genetic loci possibly related to circulating FGF21 and FGF23 levels were found. Further replication and functional studies are needed to support the present findings.

## Materials and methods

### Study population

Five thousand subjects were enrolled from Taiwan Biobank by random sampling as study population. Subjects are aged between 30 and 70 years, without cancer history and volunteered to join the Taiwan Biobank study population. Eating and drinking were refrained before blood samples were drawn. Delinked databases including biological specimens, personal data and clinical information were used in this investigation. The estimated glomerular filtration rate (eGFR) was calculated using the 4-variable equation from the Modification of Diet in Renal Disease (MDRD) Study^43^. There were only two individuals with severe renal impairment (eGFR < 30 ml/min/1.73 m^2^), and their serum FGF23 levels were 0.259 and 0.709 ng/ml.

All subjects provided written informed consent. Individuals diagnosed with diabetes mellitus (either as stated in the questionnaire or HbA1c ≥ 6.5%) were excluded.

### FGF21 and FGF23 measurements

Intact circulating FGF21 and FGF23 levels in serum of Taiwan Biobank subjects were measured using Enzyme-Linked Immunosorbent Assay (Duoset ELISA Development kit, R&D system, Inc.). The assays have high sensitivity and exhibit no interference or cross-reactivity with recombinant human FGF R1a/Fc Chimera, FGF R2a/Fc Chimera, FGF R3/Fc Chimera, FGF R4/Fc Chimera and Klotho. All standards by serial dilution were assayed in duplicates.

### Genotyping, quality control and imputation

Genome-wide genotyping of all subjects was carried out at the National Center for Genome Medicine of Academia Sinica using the Axiom-Taiwan Biobank Array Plate (TWB chip; Affymetrix Inc, Santa Clara, California)^44^. TWB chip consists of 653,291 SNPs, and was specifically customized for the Taiwanese population, who are mainly of Han-Chinese lineage, by including SNPs with detected polymorphisms in Taiwanese-based genotyping results from the Axiom Genome-Wide CHB 1 Array plate (Affymetrix Inc). SNPs from ancestry information panels, GWAS and cancer studies, as well as pharmacogenetics arrays were also incorporated into the TWB chip. PLINK (version 1.07), an open-source whole-genome association analysis toolset was used for quality control^45^. Genotypes for SNPs with batch effect were set as missing. Individuals with high missing genotype rate (> 5%), with extreme heterozygosity rate (above or below 5 standard deviations of mean heterozygosity rate) or being closely related as assessed by Identity-By-Descent (IBD) estimation (IBD ≥ 0.1875) were excluded from analyses. SNPs with high missing genotype rate (> 5%), low frequency (minor allele frequency < 1%), or deviation from Hardy–Weinberg Equilibrium (HWE) (*P* value < 10^–5^) were excluded, with 617,073 SNPs remaining. Population structure was assessed using principal component analysis. Using PLINK, we computed the principal components on an LD-pruned (*r*^2^ < 0.2) set of autosomal variants obtained by removing high-LD regions. Genome-wide genotype imputation was performed by SHAPEIT^46^ and IMPUTE2^47^ with 1,000 Genomes Project (1000GP) Phase 3 East Asian (EAS) population as reference panel. Quality control after imputation was as follows: We filtered the SNPs with imputation quality score of IMPUTE2 (info score^48^) greater than 0.3 for further analysis. Indels were removed with VCFtools^49^. SNPs with low frequency (minor allele frequency < 1%) were excluded.

### Statistical analyses

Age, body mass index (BMI), glucose level and eGFR were expressed as mean and standard deviation. FGF21 and FGF23 levels were expressed as median and interquartile range. GWAS analyses were performed using an additive genetic model in PLINK v1.07. Skewed variables including serum FGF21 and FGF23 were logarithmically transformed to approximate normal distribution. We performed regression diagnostics plots to check the assumptions of linearity, normality, and homoscedasticity, as well as to identify the influential observations for each outcome of interest (logFGF21 and logFGF23) and SNPs, adjusting for covariates. We did not find any severely violations of assumptions. Further, variance inflation factor (VIF) was used to check for multicollinearity. No correlations between independent variables were found. Linear regression was employed to analyze association between SNPs and log-transformed FGF21 and FGF23 levels. Population stratification was not obvious (λ = 1.002 for FGF21, λ = 0.999 for FGF23). Age, sex and the first ten principal components of ancestry were adjusted in Model 1, and BMI was additionally adjusted in Model 2. For log FGF23, BMI and eGFR were additionally adjusted in Model 3. The threshold for genome-wide significance was set at *P* = 5 × 10^–8^^50^. In view of the relatively small sample size and to avoid false negative results caused by too stringent threshold^51^, the *P* value threshold for suggestive results were set at 1.0 × 10^–6^^52^. Manhattan plots and quantile–quantile plots were drawn using the qqman R package^53,54^. Regional association plots were generated by LocusZoom^55^. 1,000 Genomes Project Phase 3 East Asian Ancestry was used for the reference population and Genome Reference Consortium Human Build 37 was used for gene annotation. The array-based heritability was calculated by linkage disequilibrium (LD) score regression^56^. The proportion of phenotypic variance explained by single SNP was estimated by the following formula:

$$ \frac{{{2}\beta^{{2}} {\text{*MAF*}}\left( {{1} - {\text{MAF}}} \right)}}{{{2}\beta^{{2}} *{\text{MAF}}*\left( {{1} - {\text{MAF}}} \right) + {\text{SE}}^{{2}} *{\text{2N}}*{\text{MAF}}*\left( {{1} - {\text{MAF}}} \right)}} $$

where β, SE, N, and MAF are the effect size estimate of each minor allele on the relative concentration of FGF21/ FGF23, standard error of the effect size, sample size, and minor allele frequency for the SNP, respectively^57^. Gene-based association analysis was performed using MAGMA v1.07b^14^ with LD information retrieved from 1,000 Genomes Project Phase 3 East Asian Panel. Meta-analysis pooling our study and those reported by Robinson-Cohen et al. was performed using by METAL using a random effects model ^15^.

### Bioethics statement

All methods were carried out in accordance with relevant guidelines and regulations. The experimental protocols were approved by the Institutional Review Board of National Taiwan University Hospital.

## Supplementary information

Supplementary file1

## Data Availability

Individual researchers may request to use the data for specific projects on a collaborative basis.

## References

[1] Degirolamo C, Sabba C, Moschetta A (2016). Therapeutic potential of the endocrine fibroblast growth factors FGF19, FGF21 and FGF23. Nat. Rev. Drug Discov..

[2] Badman MK (2007). Hepatic fibroblast growth factor 21 is regulated by PPARalpha and is a key mediator of hepatic lipid metabolism in ketotic states. Cell Metab..

[3] Bookout AL (2013). FGF21 regulates metabolism and circadian behavior by acting on the nervous system. Nat. Med..

[4] Chartoumpekis DV (2011). Brown adipose tissue responds to cold and adrenergic stimulation by induction of FGF21. Mol. Med..

[5] Wente W (2006). Fibroblast growth factor-21 improves pancreatic beta-cell function and survival by activation of extracellular signal-regulated kinase 1/2 and Akt signaling pathways. Diabetes.

[6] Owen BM (2014). FGF21 acts centrally to induce sympathetic nerve activity, energy expenditure, and weight loss. Cell Metab..

[7] Dutchak PA (2012). Fibroblast growth factor-21 regulates PPARgamma activity and the antidiabetic actions of thiazolidinediones. Cell.

[8] Kharitonenkov A, Adams AC (2014). Inventing new medicines: The FGF21 story. Mol. Metab..

[9] Kanbay M (2017). Novel faces of fibroblast growth factor 23 (FGF23): iron deficiency, inflammation, insulin resistance, left ventricular hypertrophy, proteinuria and acute kidney injury. Calcif. Tissue Int..

[10] Angelin B, Larsson TE, Rudling M (2012). Circulating fibroblast growth factors as metabolic regulators–a critical appraisal. Cell Metab..

[11] Mirza MA (2011). Circulating fibroblast growth factor-23 is associated with fat mass and dyslipidemia in two independent cohorts of elderly individuals. Arterioscler. Thromb. Vasc. Biol..

[12] Kinoshita Y, Fukumoto S (2018). X-linked hypophosphatemia and FGF23-related hypophosphatemic diseases: prospect for new treatment. Endocr. Rev..

[13] Robinson-Cohen C (2018). Genetic variants associated with circulating fibroblast growth factor 23. J. Am. Soc. Nephrol..

[14] de Leeuw CA, Mooij JM, Heskes T, Posthuma D (2015). MAGMA: generalized gene-set analysis of GWAS data. PLoS Comput. Biol..

[15] Willer CJ, Li Y, Abecasis GR (2010). METAL: fast and efficient meta-analysis of genomewide association scans. Bioinformatics.

[16] Chatterjee TK, Liu Z, Fisher RA (2003). Human RGS6 gene structure, complex alternative splicing, and role of N terminus and G protein gamma-subunit-like (GGL) domain in subcellular localization of RGS6 splice variants. J. Biol. Chem..

[17] Uhlen M (2015). Proteomics. Tissue-based map of the human proteome. Science.

[18] Dohlman HG, Thorner J (1997). RGS proteins and signaling by heterotrimeric G proteins. J. Biol. Chem..

[19] Pashkov V (2011). Regulator of G protein signaling (RGS16) inhibits hepatic fatty acid oxidation in a carbohydrate response element-binding protein (ChREBP)-dependent manner. J. Biol. Chem..

[20] Stewart A (2015). Regulator of G protein signaling 6 is a critical mediator of both reward-related behavioral and pathological responses to alcohol. Proc. Natl. Acad. Sci. USA.

[21] Perry KD (2017). Composite hemangioendothelioma with neuroendocrine marker expression: an aggressive variant. Mod. Pathol..

[22] Meng W, Deshmukh HA, Donnelly LA (2015). A genome-wide association study provides evidence of sex-specific involvement of Chr1p35.1 (ZSCAN20-TLR12P) and Chr8p23.1 (HMGB1P46) With Diabetic Neuropathic Pain. EbioMedicine.

[23] Zhang D, Jiang P, Xu Q, Zhang X (2011). Arginine and glutamate-rich 1 (ARGLU1) interacts with mediator subunit 1 (MED1) and is required for estrogen receptor-mediated gene transcription and breast cancer cell growth. J. Biol. Chem..

[24] Sigurdsson S (2017). Sequence variants in ARHGAP15, COLQ and FAM155A associate with diverticular disease and diverticulitis. Nat. Commun..

[25] Lagace TA (2014). PCSK9 and LDLR degradation: regulatory mechanisms in circulation and in cells. Curr. Opin. Lipidol..

[26] White KE (2001). Autosomal-dominant hypophosphatemic rickets (ADHR) mutations stabilize FGF-23. Kidney Int..

[27] Benet-Pages A (2004). FGF23 is processed by proprotein convertases but not by PHEX. Bone.

[28] Kato K (2006). Polypeptide GalNAc-transferase T3 and familial tumoral calcinosis. Secretion of fibroblast growth factor 23 requires O-glycosylation. J. Biol. Chem..

[29] Jones EY, Fugger L, Strominger JL, Siebold C (2006). MHC class II proteins and disease: a structural perspective. Nat. Rev. Immunol..

[30] Feger M (2017). The production of fibroblast growth factor 23 is controlled by TGF-beta2. Sci. Rep..

[31] Qiu T (2010). TGF-beta type II receptor phosphorylates PTH receptor to integrate bone remodelling signalling. Nat. Cell. Biol..

[32] Zerr P (2015). Vitamin D receptor regulates TGF-beta signalling in systemic sclerosis. Ann. Rheum. Dis..

[33] Subramaniam N (2001). Cross-talk between 1,25-dihydroxyvitamin D3 and transforming growth factor-beta signaling requires binding of VDR and Smad3 proteins to their cognate DNA recognition elements. J. Biol. Chem..

[34] Leslie R, O'Donnell CJ, Johnson AD (2014). GRASP: analysis of genotype-phenotype results from 1390 genome-wide association studies and corresponding open access database. Bioinformatics.

[35] Need AC (2009). A genome-wide investigation of SNPs and CNVs in schizophrenia. PLoS Genet..

[36] Kathiresan S (2009). Common variants at 30 loci contribute to polygenic dyslipidemia. Nat. Genet..

[37] Teslovich TM (2010). Biological, clinical and population relevance of 95 loci for blood lipids. Nature.

[38] Lango Allen H (2010). Hundreds of variants clustered in genomic loci and biological pathways affect human height. Nature.

[39] Stambolian D (2013). Meta-analysis of genome-wide association studies in five cohorts reveals common variants in RBFOX1, a regulator of tissue-specific splicing, associated with refractive error. Hum. Mol. Genet..

[40] Pankratz N (2012). Meta-analysis of Parkinson's disease: identification of a novel locus, RIT2. Ann. Neurol..

[41] Deng G (2018). The value of older donors' klotho level in predicting recipients' short-term renal function. Med. Sci. Monit..

[42] Farias-Basulto A (2018). Circulating levels of soluble klotho and fibroblast growth factor 23 in diabetic patients and its association with early nephropathy. Arch. Med. Res..

[43] Levey AS (2006). Using standardized serum creatinine values in the modification of diet in renal disease study equation for estimating glomerular filtration rate. Ann. Intern. Med..

[44] Chen CH (2016). Population structure of Han Chinese in the modern Taiwanese population based on 10,000 participants in the Taiwan Biobank project. Hum. Mol. Genet..

[45] Purcell S (2007). PLINK: a tool set for whole-genome association and population-based linkage analyses. Am. J. Hum. Genet..

[46] Delaneau O, Zagury JF, Marchini J (2013). Improved whole-chromosome phasing for disease and population genetic studies. Nat. Methods.

[47] Howie B, Fuchsberger C, Stephens M, Marchini J, Abecasis GR (2012). Fast and accurate genotype imputation in genome-wide association studies through pre-phasing. Nat. Genet..

[48] Marchini J, Howie B (2010). Genotype imputation for genome-wide association studies. Nat. Rev. Genet..

[49] Danecek P (2011). The variant call format and VCFtools. Bioinformatics.

[50] Pe'er I, Yelensky R, Altshuler D, Daly MJ (2008). Estimation of the multiple testing burden for genomewide association studies of nearly all common variants. Genet. Epidemiol..

[51] Fallin MD, Kao WH (2011). Is, "X"-WAS the future for all of epidemiology?. Epidemiology.

[52] Duggal P, Gillanders EM, Holmes TN, Bailey-Wilson JE (2008). Establishing an adjusted p-value threshold to control the family-wide type 1 error in genome wide association studies. BMC Genom..

[53] Turner, S. *qqman: An R Package for Visualizing GWAS Results Using Q-Q and Manhattan Plots*. (2014).

[54] Team, R. D. C. *R: A Language and Environment for Statistical Computing*. (2008).

[55] Pruim RJ (2010). LocusZoom: regional visualization of genome-wide association scan results. Bioinformatics.

[56] Bulik-Sullivan BK (2015). LD Score regression distinguishes confounding from polygenicity in genome-wide association studies. Nat. Genet..

[57] Shim H (2015). A multivariate genome-wide association analysis of 10 LDL subfractions, and their response to statin treatment, in 1868 Caucasians. PLoS ONE.

