# Supplementary material for: Genome-wide association study for circulating fibroblast growth factor 21 and 23
Source: Sci Rep. 2020 Sep 3;10:14578. doi: 10.1038/s41598-020-71569-8 (PMC7471933; doi:10.1038/s41598-020-71569-8)
Supplement: Supplementary file 1 — Supplementary file1 [file 41598_2020_71569_MOESM1_ESM.pdf]

Full title:

Genome-wide association study for circulating fibroblast growth factor 21 and 23

Short title: GWAS for FGF21 and FGF23

Gwo-Tsann Chuang[1,2*],Pi-Hua Liu[3,4*], Tsui-Wei Chyan[5], Chen-Hao Huang[5], Yu-Yao Huang[4,6],Chia-Hung Lin[4,7],Jou-Wei Lin[8],Chih-Neng Hsu[8], Ru-YiTsai[8],Meng-Lun Hsieh[9], Siao-Lin Lee[9], Wei-shun Yang[2,9], Cassianne Robinson-Cohen[10], Chia-Ni Hsiung[11], Chen-Yang Shen[12,13],Yi-Cheng Chang[2,9,12]

1. Department of Pediatrics, National Taiwan University Hospital, College of Medicine, National Taiwan University, Taipei, Taiwan

2. Graduate Institute of Medical Genomics and Proteomics, NationalTaiwanUniversity, Taipei, Taiwan

3. Clinical Informatics and MedicalStatisticsResearchCenter, College of Medicine, ChangGungUniversity, Taoyuan, Taiwan

4. Division of Endocrinology and Metabolism, Department of Internal Medicine, ChangGungMemorialHospital at Linkou, Taoyuan, Taiwan

5. Institute of Molecular Medicine, College of Medicine, NationalTaiwanUniversity, Taipei, Taiwan

6. Department of Medical Nutrition Therapy, ChangGungMemorialHospital at Linkou, Taoyuan, Taiwan

7. Department of Chinese Medicine, College of Medicine, ChangGungUniversity, Taoyuan, Taiwan

8. Cardiovascular Center, NationalTaiwanUniversityHospital Yun-Lin Branch

9. Department of Internal Medicine, College of Medicine, NationalTaiwanUniversity, Taipei, Taiwan

10. Division of Nephrology, Department of Medicine, Vanderbilt University Medical Center, Nashville, Tennessee, United States

11. Data Science Statistical Cooperation Center, Institute of Statistical Science, Academia Sinica, Taipei, Taiwan

12. Institute of Biomedical Sciences, Academia Sinica, Taipei, Taiwan

13. College of Public Health, China Medical University, Taichung, Taiwan

* Equal contributing co-first authors

Corresponding author: Yi-Cheng Chang (M.D., Ph.D.)

5F, No.2, Xuzhou Rd., Zhongzheng Dist.,

Taipei 100, Taiwan

Email: b83401040@gmail.com

Phone: +886-2-2312-3456 Ext. 88656

SupplementaryTableS1. Top genetic polymorphisms associated with log-transformed FGF23 level (removal of 2 individuals with eGFR < 30 ml/min/1.73m^2)

SNP	Chr	Position	Nearest gene	FGF23 increasing allele	Other allele	FGF23 increasing allele frequency	Model 1		Model 2		Model 3	
							P value	Beta (SEM)	P value	Beta (SEM)	P value	Beta (SEM)
rs17111495	1	55500706	*PCSK9*	G	C	0.98	1.05 x 10^{-10}	0.463 (0.071)	1.59 x 10^{-10}	0.458 (0.071)	1.57 x 10^{-10}	0.458 (0.071)
rs17843626	6	32621013	*HLA-DQA1*	G	A	0.49	1.82 x 10^{-8}	0.117 (0.021)	1.72 x 10^{-8}	0.117 (0.021)	1.77 x 10^{-8}	0.117 (0.021)
rs2798631	1	218611878	*TGFB2*	G	A	0.75	4.75 x 10^{-7}	0.115 (0.023)	3.58 x 10^{-7}	0.116 (0.023)	3.56 x 10^{-7}	0.116 (0.023)

Model 1 wasadjusted for age, sex and the first ten principal components of ancestry. Model 2 was additionally adjusted for BMI. Model 3 was additionally adjusted for BMI and eGFR. SNP, single nucleotide polymorphism; Chr, chromosome.

Supplementary Table S2. Association of genetic polymorphisms identified in previous GWAS for FGF23 level for European ancestry in Taiwan biobank

SNP	Chr	Position	Nearest gene	FGF23 increasing allele	Other allele	FGF23 increasing allele frequency	P value	Beta (SEM)
rs17216707	20	52732362	*CYP24A1*	C	T	0.02	0.640	0.033 (0.071)
rs2769071	9	136145974	*ABO*	A	G	0.65	0.202	0.026 (0.021)
rs11741640	5	176792743	*RGS14*	A	G	0.13	0.157	0.043 (0.030)
rs17479566	9	71198014	*LINC01506*	C	T	0.71	0.930	0.002 (0.022)
rs9925837	16	79927303	*LINC01229*	A	G	0.82	0.288	0.028 (0.026)

Effect estimate adjusted for age, sex and first ten principal components of ancestry. SNP, single nucleotide polymorphism; Chr, chromosome.

Supplementary Table S3. Meta-analysis of loci associated with FGF23 level found in our study

SNP	Chr	Position	Nearest gene	FGF23 increasing allele	Other allele	Taiwan (N=4201)			European (N=16624)			Meta-analysis (N=20825)	
						FGF23 increasing allele frequency	P value	Beta (SEM)	FGF23 increasing allele frequency	P value	Beta (SEM)	Z score	P value
rs17111495	1	55500706	*PCSK9*	G	C	0.98	1.04×10^{-10}	0.463 (0.071)	0.93	0.612	0.31 (0.61)	3.355	7.92×10^{-4}
rs17843626	6	32621013	*HLA-DQA1*	G	A	0.49	1.80×10^{-8}	0.117 (0.021)	0.70	0.224	0.19 (0.15)	3.344	8.27×10^{-4}
rs2798631	1	218611878	*TGFB2*	G	A	0.75	4.97×10^{-7}	0.115 (0.023)	0.49	0.261	0.46 (0.41)	3.533	4.11×10^{-4}

Effect estimate adjusted for age, sex and first ten principal components of ancestry. SNP, single nucleotide polymorphism; Chr, chromosome. All 3 SNPs had the same direction of effect among Taiwan and European population.

Supplementary Figure S1. Regional association plots of log FGF23 at five loci previously found by Robinson-Cohen and colleagues.

(a) rs17216707

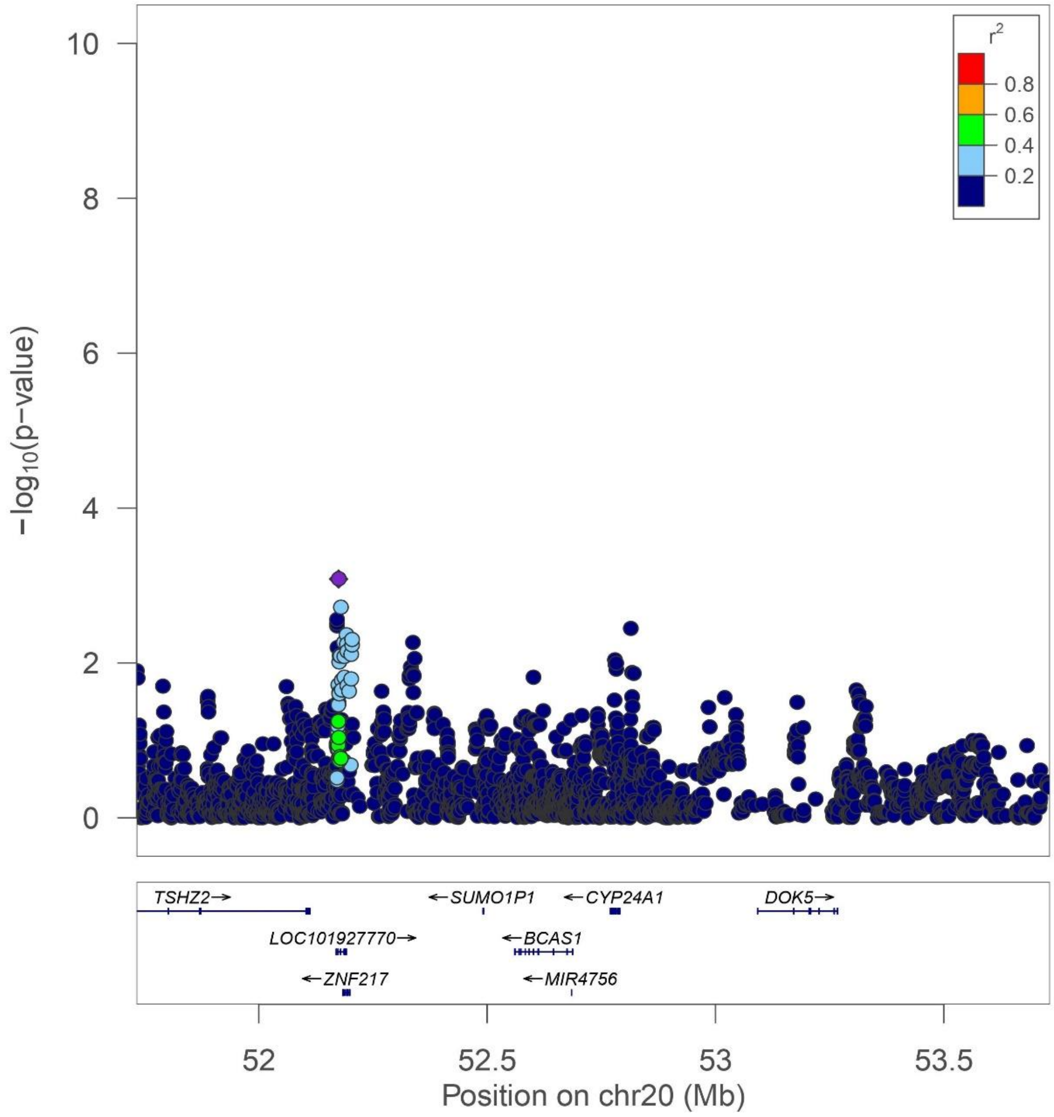

(b) rs2769071

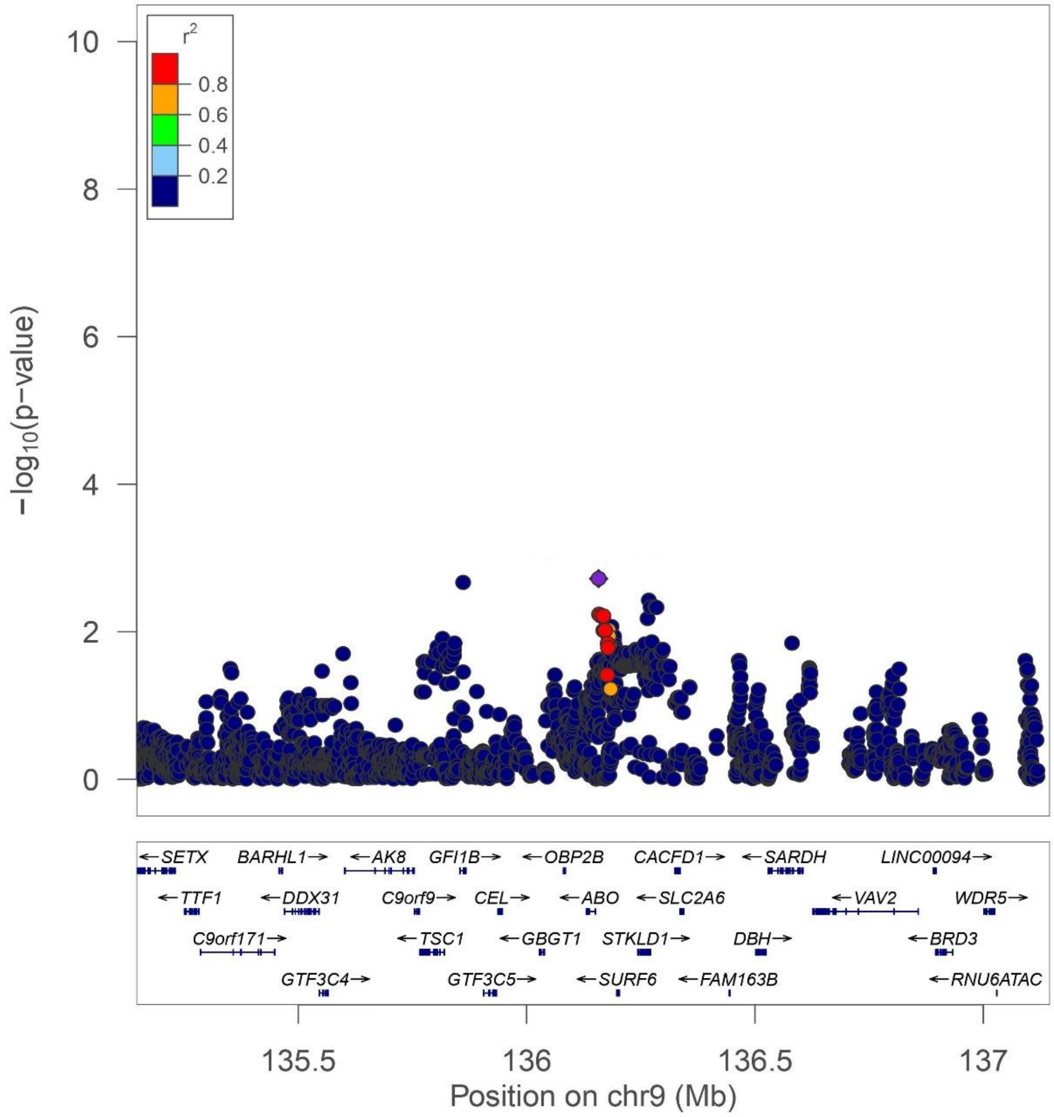
r2
0.8
0.6
0.4
0.2
10
8
6
4
2
0
−log10(p−value)
←SETX
BARHL1→
←AK8
GFI1B→
←OBP2B
CACFD1→
←SARDH
LINC00094→
←TTF1
←DDX31
C9orf9→
CEL→
←ABO
←SLC2A6
←VAV2
WDR5→
C9orf171→
←TSC1
←GBGT1
STKLD1→
DBH→
←BRD3
GTF3C4→
GTF3C5→
←SURF6
←FAM163B
←RNU6ATAC
135.5
136
136.5
137
Position on chr9 (Mb)

(c) rs11741640

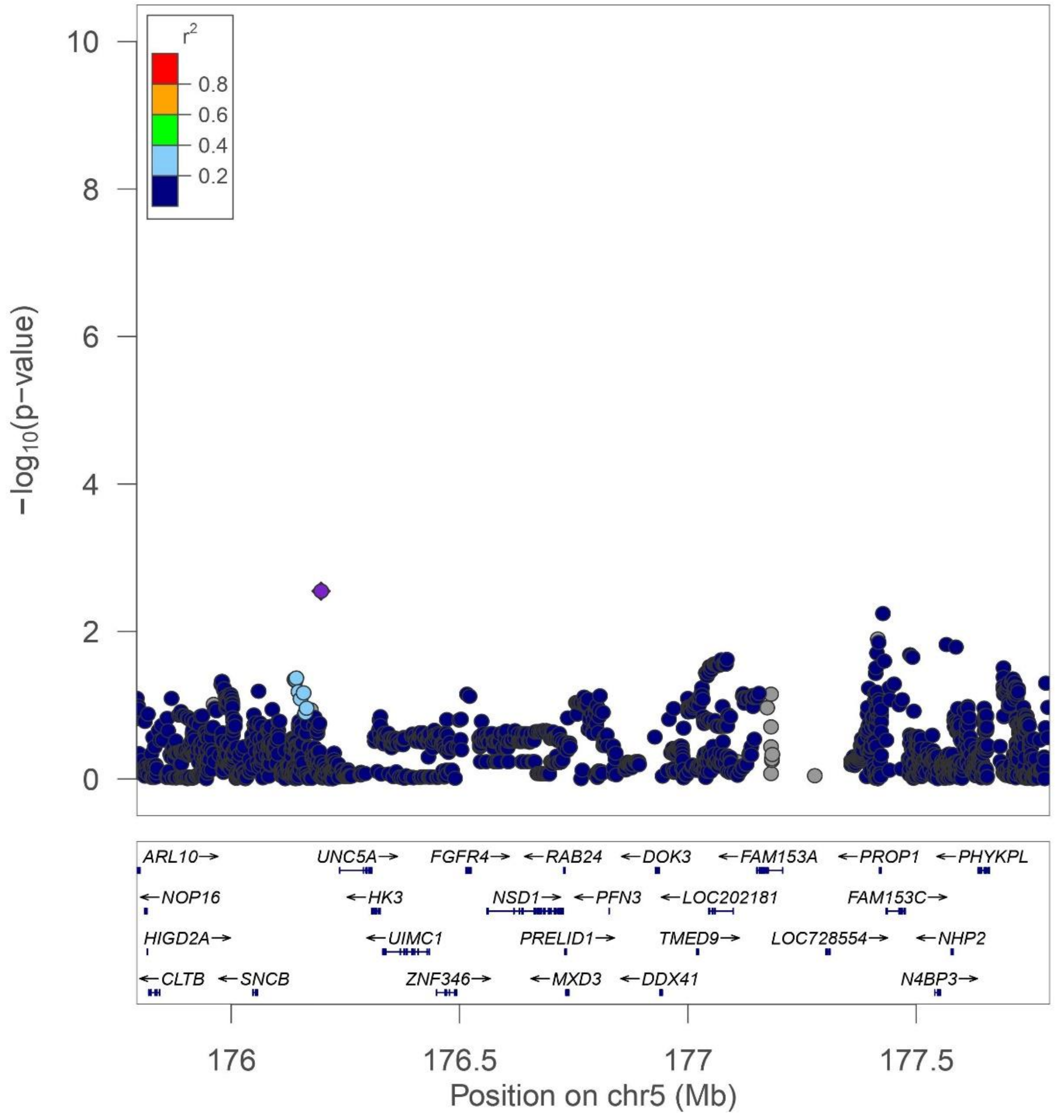

(d) rs17479566

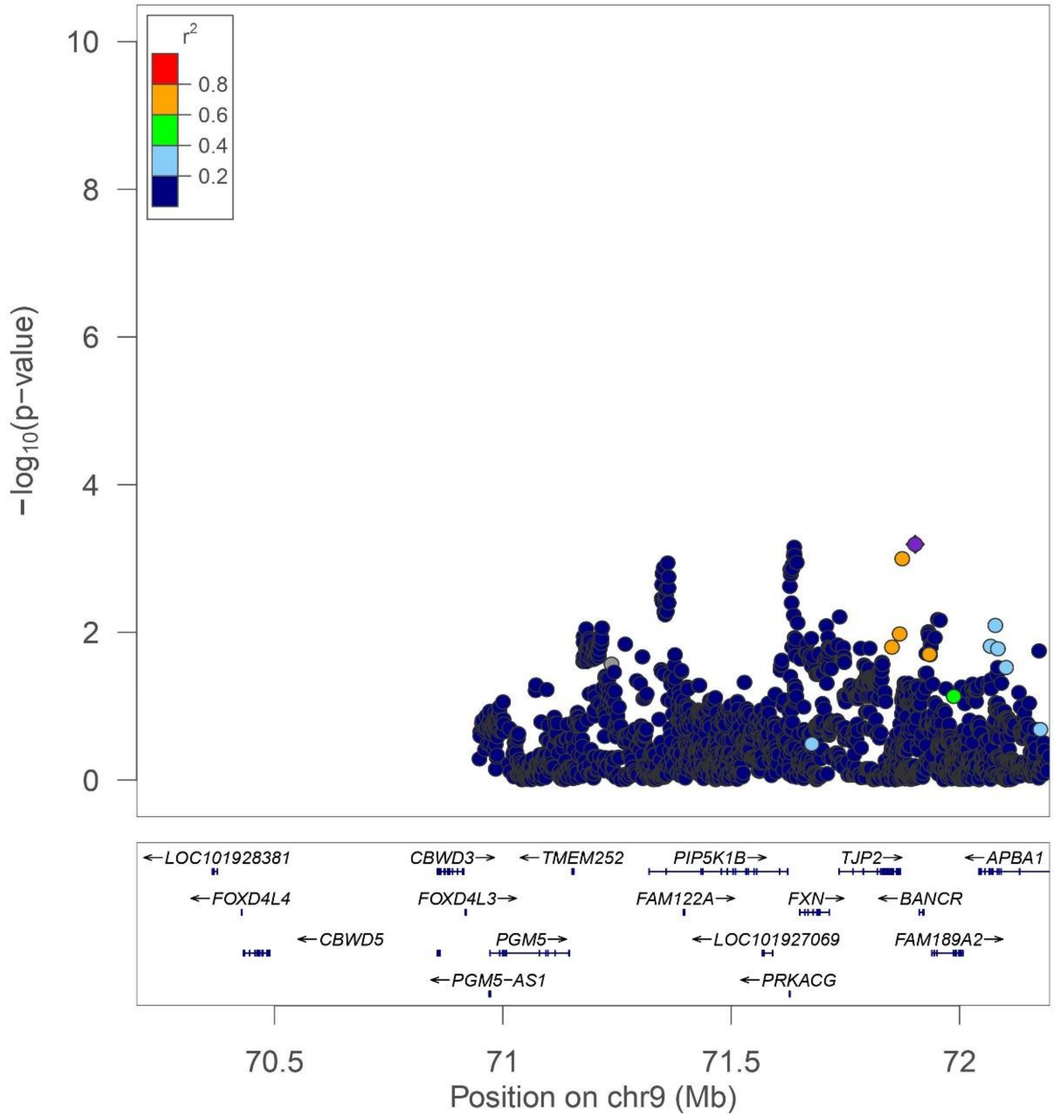

(e) rs9925837

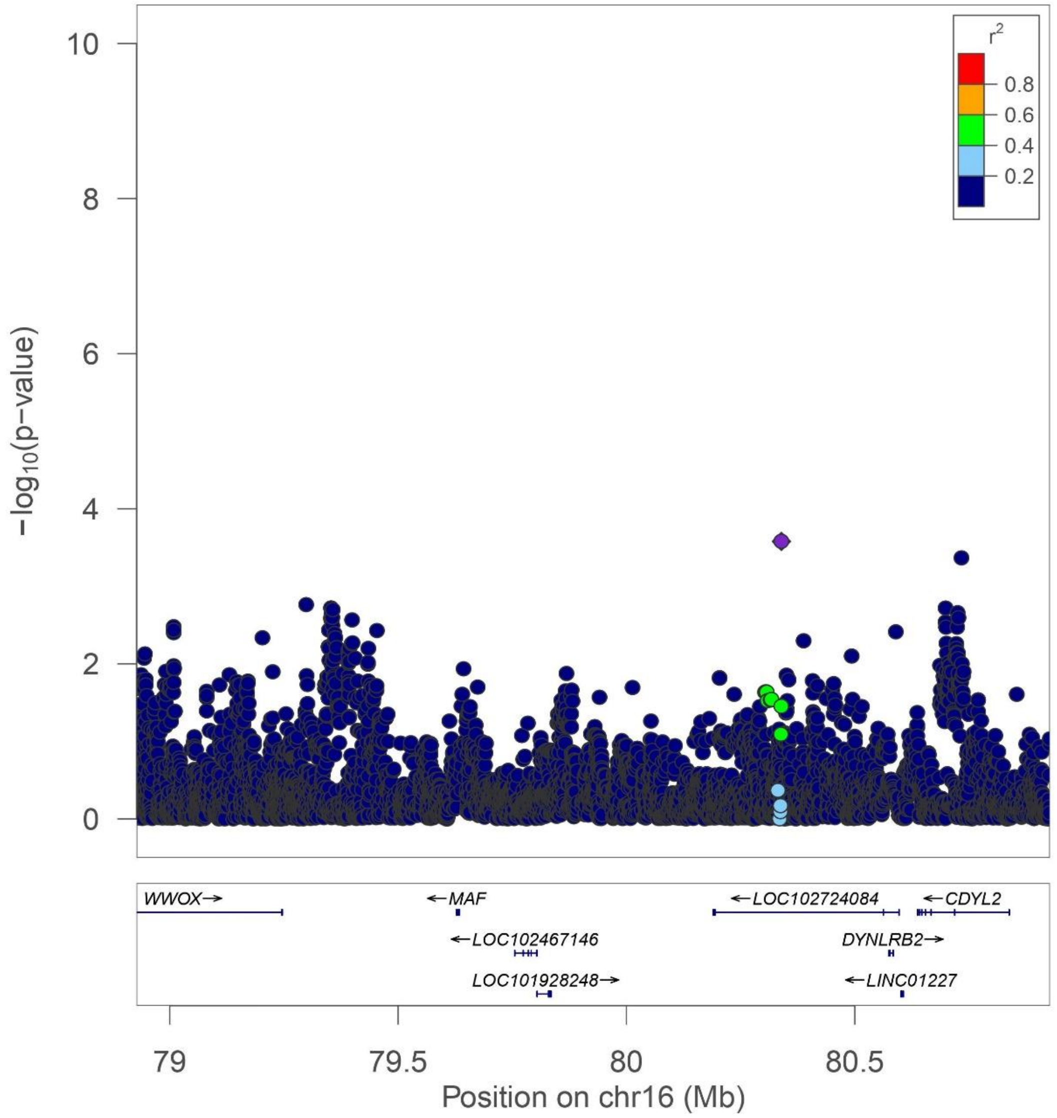
r2
0.8
0.6
0.4
0.2
10
8
6
4
2
0
−log10(p−value)
WWOX→
←MAF
←LOC102467146
LOC101928248→
←LOC102724084
←CDYL2
DYNLRB2→
←LINC01227
79
79.5
80
80.5
Position on chr16 (Mb)
